# Supplementary material for: Analysis of the Properties of 44 ABC Transporter Genes from Biocontrol Agent Trichoderma asperellum ACCC30536 and Their Responses to Pathogenic Alternaria alternata Toxin Stress
Source: Curr Issues Mol Biol. 2023 Feb 12;45(2):1570–86. doi: 10.3390/cimb45020101 (PMC9955796; doi:10.3390/cimb45020101)
Supplement: Supplementary file 1 [file cimb-45-00101-s001.zip › cimb-2103677-supplementary.pdf]

Table S1 Properties of the 44 ABC transporters in *Trichoderma asperellum*.

Gene name	Location of gene on chromosome	Protein ID	NE	NI	AA	pI	MW (kDa)	AOCD	Multidomain	Conserved Domain
ABC01	scaffold_5:1330229-1335406(+)	26122	2	1	1698	5.7	187.56	A-B-A-B	MRP	324-659,707-933,1156-1356,1403-1660
ABC02	scaffold_3:1602050-1606909(-)	24492	1	0	1619	8.3	176.23	B-C-B	Rim protein	471-694,1016-1137,1267-1482
ABC03	scaffold_6:1245891-1250899(+)	57754	1	0	1601	7.27	174.31	B-E-B	Rim protein	464-687,1003-1154,1250-1465
ABC04	scaffold_7:566523-571800(+)	193557	4	3	1580	6.09	174.27	A-B-A-B	CFTR	418-584,630-858,1033-1265,1153-1309
ABC05	scaffold_15:669827-674943(+)	51252	4	3	1156	7.56	173.28	A-B-A-B	MRP	317-590,638-840,988-

										1257,1304-1537
ABC06	scaffold_11:638450-643615(+)	173750	5	4	1566	5.79	172.37	A-B-A-B	MdlB	291-582,621-845,1010-1253,1295-1539
ABC07	scaffold_2:2877202-2882351(+)	159930	3	2	1250	6.12	168.32	A-B-A-B	CFTR	315-582,627-827,921-1161,1249-1494
ABC08	scaffold_9:637077-643406(-)	59384	15	14	1528	5.91	167.1	A-B-A-B	CFTR	281-556,657-855,946-1193,1280-1511
ABC09	scaffold_8:1749604-1754389(+)	38146	3	2	1439	9.75	164.8	A-B-A-B	MRP	295-431,599-806,903-1139,1207-1456
ABC10	scaffold_6:1669360-1674235(-)	457746	15	14	1493	5.92	163.86	A-B-A-B	MRP	289-541,598-803,893-

										1090,1211-1477
ABC11	scaffold_5:371144-376301(-)	190301	3	2	1470	6.43	163.55	A-B-A-B	CFTR	195-514,608-798,893-1160,1202-1430
ABC12	scaffold_5:2335370-2340344(-)	166239	8	7	1454	6.6	161.54	B-A-B	MRP	591-796,879-1077,1197-1438
ABC13	scaffold_5:770479-775940(-)	78932	16	15	1462	7.3	161.41	A-B-A-B	MRP	287-553,625-830,912-1161,1212-1434
ABC14	scaffold_2:2042620-2048126(+)	65153	15	14	1472	7.35	160.83	A-B-A-B	MRP	281-551,630-830,918-1182,1229-1449
ABC15	scaffold_11:93761-99715(+)	60292	15	14	1446	6.61	159.11	A-B-A-B	MRP	279-531,613-809,886-1089,1197-1414

ABC16	scaffold_1:325664-329929(+)	63327	1	0	1421	6.77	158	A-B-A-B	MRP	392-480,606-807,929-1101,1202-1414
ABC17	scaffold_3:2326294-2331426(+)	187370	15	14	1413	7.76	155.93	A-B-A-B	CFTR	250-521,582-779,870-1116,1169-1405
ABC18	scaffold_4:1219886-1224871(+)	188812	3	2	1367	6.33	149.67	A-B-A-B	MDR	138-408,464-710,796-1076,1126-1365
ABC19	scaffold_3:1099634-1103753(-)	24350	2	1	1346	6.8	149.25	B-C-B-C	Protein white	107-313,401-613,718-949,1076-1275
ABC20	scaffold_12:640384-644759(-)	39709	3	2	1341	7.49	146.72	A-B-A-B	MdlB	77-323,372-610,770-1044,1090-1339

ABC21	scaffold_6:623567-627580(-)	139504	1	0	1337	6.41	146.39	A-B-A-B	MDR	104-385,432-678,769-1048,1097-1335
ABC22	scaffold_29:17508-21673(+)	154395	3	2	1337	6.49	146.11	A-B-A-B	MDR	102-381,429-674,773-1052,1096-1335
ABC23	scaffold_1:2903039-2907805(+)	64176	12	11	1310	6.2	144.84	A-B-A-B	MDR	66-347,392-683,749-1019,1072-1310
ABC24	scaffold_11:1091827-1096466(+)	60557	9	8	1289	5.97	140.56	A-B-A-B	MDR	80-342,393-638,716-990,1039-1290
ABC25	scaffold_1:100127-104858(-)	155244	11	10	1261	6.67	138.37	A-B-A-B	MDR	71-327,372-635,704-974,1027-1261
ABC26	scaffold_6:108438-112752(+)	395259	3	2	1280	6.33	138.01	A-B-A-B	MDR	45-270,325-605,697-968,1041-1281

ABC27	scaffold_8:1063376-1067657(+)	195530	5	4	1275	6.36	137.75	A-B-A-B	MDR	47-326,373-619,700-981,1037-1276
ABC28	scaffold_6:298511-302824(+)	138427	7	6	1266	5.83	137.13	A-B-A-B	CFTR	95-357,400-596,693-899,1009-1234
ABC29	scaffold_6:792799-796903(-)	192275	2	1	1113	6.2	122.48	B-B	UuP	488-676,727-1021
ABC30	scaffold_12:686978-690799(-)	60895	4	3	1077	5.48	117.17	B-C	Protein white	369-596,808-1018
ABC31	scaffold_4:2162032-2166124(+)	35803	3	2	1055	5.77	117.13	B-B-B	UuP	446-632,682-985,713-795
ABC32	scaffold_6:571499-574783(+)	46574	3	2	966	9.87	110.08	A-B	ATM1	360-637,683-918
ABC33	scaffold_4:2335986-2339176(+)	67305	7	6	935	8.26	103.06	A-B	MdlB	284-553,599-834

ABC34	scaffold_17:462008-464428(-)	51699	1	0	806	9.43	86.93	A-B	MdlB	188-456,510-749
ABC35	scaffold_4:404984-407606(-)	55941	3	2	749	5.71	82.72	B-D-B	UuP	201-442,436-522,553-725
ABC36	scaffold_2:2113968-2116435(+)	129097	5	4	737	5.92	81.86	B-B-D-B	UuP	83-161,262-365,361-432,489-684
ABC37	scaffold_1:3104696-3107034(+)	127572	4	3	710	9.41	81.01	A-B	3a0123	90-371,468-674
ABC38	scaffold_3:1180302-1182933(-)	161063	2	1	728	9.4	79.61	A-B	ATM1	146-422,469-706
ABC39	scaffold_25:29785-32063(+)	84850	2	1	655	5.93	73.83	A-B	ATM1	174-375,421-656
ABC40	scaffold_16:47359-49428(-)	151508	3	2	631	7.2	69.76	B-C	Protein white	34-266,356-564

ABC41	scaffold_43:7456-9454(+)	154872	3	2	619	8.14	69.56	B-C	Protein white	25-253,342-545
ABC42	scaffold_17:13345-15352(-)	31428	3	2	620	6.17	69.09	B-D-B	UuP	88-306,304-392,399-595
ABC43	scaffold_16:35781-38167(+)	73462	5	4	616	9.37	66.05	A-B	MdlB	109-325,376-619
ABC44	scaffold_8:1897631-1899321(-)	143077	5	4	491	8.79	55.33	A-B	ATM1	11-196,250-483

Note: NE: Number of exon; NI: Number of intron; AA: Number of amino acids; pI: Isoelectric poin; MW: molecular weight; AOCD: Arrangement of conserved domain.
